# A phase I radiation dose-escalation study to determine the maximal dose of radiotherapy in combination with weekly gemcitabine in patients with locally advanced pancreatic adenocarcinoma

**DOI:** 10.1186/1748-717X-3-30

**Published:** 2008-09-22

**Authors:** Tom Budiharto, Karin Haustermans, Eric Van Cutsem, Werner Van Steenbergen, Baki Topal, Raymond Aerts, Nadine Ectors, Didier Bielen, Dirk Vanbeckevoort, Laurence Goethals, Chris Verslype

**Affiliations:** 1 Department of Radiotherapy, University Hospitals Leuven, Leuven, Belgium; 2 Department of Abdominal Surgery, University Hospitals Leuven, Leuven, Belgium; 3 Department of Radiology, University Hospitals Leuven, Leuven, Belgium; 4 Department of Pathology, University Hospitals Leuven, Leuven, Belgium; 5 Department of Gastroenterology, University Hospitals Leuven, Leuven, Belgium; 6 Leuvens Kanker Instituut, LKI, Leuven, Belgium

## Abstract

**Background:**

The primary objective of this study was to determine the maximum tolerated dose (MTD) of escalating doses of radiotherapy (RT) concomitantly with a fixed dose of gemcitabine (300 mg/m^2^/week) within the same overall treatment time.

**Methods:**

Thirteen patients were included. Gemcitabine 300 mg/m^2^/week was administered prior to RT. The initial dose of RT was 45 Gy in 1.8 Gy fractions, escalated by adding 5 fractions of 1.8 Gy (one/week) to a dose of 54 Gy with a total duration kept at 5 weeks. All patients received a dynamic MRI to assess the pancreatic respiratory related movements. Toxicity was scored using the RTOG-EORTC toxicity criteria.

**Results:**

Three of six patients experienced an acute dose limiting toxicity (DLT) at the 54 Gy dose level. For these patients a grade III gastro-intestinal toxicity (GI) was noted. Patients treated at the 45 Gy dose level tolerated therapy without DLT. The 54 Gy dose level was designated as the MTD and was deemed not suitable for further investigation.

Between both dose levels, there was a significant difference in percentage weight loss (p = 0.006) and also in cumulative GI toxicity (p = 0.027). There was no grade 3 toxicity in the 45 Gy cohort versus 4 grade 3 toxicity events in the 54 Gy cohort. The mean dose to the duodenum was significantly higher in the 54 Gy cohort (38.45 Gy vs. 51.82 Gy; p = 0.001).

**Conclusion:**

Accelerated dose escalation to a total dose of 54 Gy with 300 mg/m^2^/week gemcitabine was not feasible. GI toxicity was the DLT. Retrospectively, the dose escalation of 9 Gy by accelerated radiotherapy might have been to large. A dose of 45 Gy is recommended. Considering the good patient outcomes, there might be a role for the investigation of a fixed dose of gemcitabine and concurrent RT with small fractions (1.8 Gy/day) in borderline resectable or unresectable non-metastatic locally advanced pancreatic cancer.

## Background

Pancreatic ductal adenocarcinoma has a 5-year survival rate of 0.4% [1] to 5% [2]. Because of this dismal prognosis, it is one of the top four causes of cancer death in the Western world [3]. Surgical resection of the tumour is associated with improved 5-year survival up to approximately 20% [4], but unfortunately, only 10% to 20% of patients are candidate for surgery at initial diagnosis [5] and there remains a high incidence of local tumour recurrence [6]. Approximately 40% of patients with pancreatic cancer present with locally advanced non-metastatic disease. Tumour adherence or invasion into adjacent structures, particularly the celiac and superior mesenteric vasculature (T3-4 or stage III disease according to the TNM-classification) make complete resection difficult or impossible. The median survival of patients with non-metastatic locally advanced pancreatic cancer (LAPC) varies between 6 to 12 months when treated with palliative therapy.

Based on an early trial by the Gastrointestinal Tumour Study Group, which demonstrated a modest survival benefit with chemoradiotherapy (CRT) when compared to radiation therapy (RT) or chemotherapy alone, patients who have unresectable disease are often treated with concurrent fluorouracil (5-FU)-based CRT [7].

Gemcitabine has been shown to provide a survival advantage over 5-FU in patients with locally advanced (unresectable) or metastatic pancreatic cancer [8]. Also, different in vitro and in vivo studies have demonstrated that gemcitabine is a potent radiosensitizer in human cancer cell lines including pancreatic cancer cell lines [9-12]. Thus integration of gemcitabine with radiation in a CRT protocol represents an alternative approach to improve outcome in patients with pancreatic cancer [13].

Based on studies of hyperfractionation and/or acceleration in squamous cell cancer of head and neck [14], one could expect that the combination of RT dose escalation and concurrent gemcitabine would also improve the rate of loco-regional control and in the same time overall survival in patients with non-metastatic LAPC.

The primary objective of this study was to define the maximum tolerated dose (MTD) of escalating doses of RT delivered concurrently with a fixed dose of gemcitabine (300 mg/m^2^) administered on a weekly basis within the same overall treatment time in patients with borderline resectable or unresectable LAPC.

## Methods

### Eligibility

Eligibility criteria for study entry included cytological or histological confirmation of pancreatic adenocarcinoma. Patients were required to have T3-4 disease (Tumour adherence or invasion into adjacent structures, particularly the celiac and superior mesenteric vasculature), N0-1 according to the TNM-classification, without distant metastases (M0). Eligible patients were required to be ≥ 18 years, to have a WHO performance status ≤ 2 and a life expectancy of more than 3 months. Pre-treatment evaluation included a complete history and physical examination, a diagnostic CT scan of the abdomen with intravenous (IV) contrast, as well as a blood exam with an adequate haematological (absolute neutrophil count (ANC) ≥ 1.5 × 103/L, platelets > 100 × 103/L, hemoglobin level > 10 g/dL), renal (serum creatinin concentration < 2 mg/dL) and liver function (bilirubin ≤ 1.5 times UNL, SGOT and SGPT ≤ 2.5 times UNL). All patients underwent an ERCP and also a laparoscopy to exclude peritoneal metastasis. Patients were excluded for any other concomitant cancers or serious illnesses (medical or psychiatric) and for metastatic disease. This phase I trial was approved by an independent ethics committee and all patients gave written informed consent before study enrolment.

### Study design

**Figure 1:**
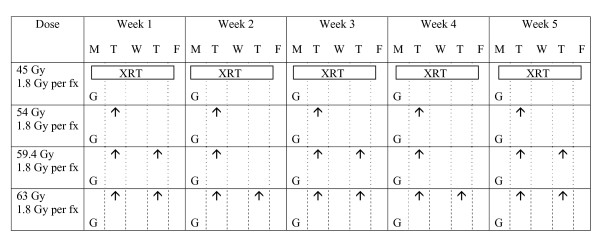
**Treatment scheme: different RT dose levels to be investigated**. Radiotherapy dose escalation scheme investigated in this phase I trial; gemcitabine (G) was administered on day 1 of week 1, 2, 3, 4 and 5 in a single dose of 300 mg/m^2 ^as a 30-min IV infusion at least one hour prior to RT; the starting dose of RT was 45 Gy in 25 fractions of 1.8 Gy per day (XRT); dose escalation was achieved by giving 2 fractions of 1.8 Gy per day on a fixed day, with an interfraction interval of at least 6 hours, an arrow indicates an extra fraction of 1.8 Gy on that day; the escalating dose levels tested were: 54 Gy, 59.4 Gy and 63 Gy; the total duration of the RT was kept on 5 weeks.

The treatment schedule is shown in Figure 1. Gemcitabine was administered weekly in a single dose of 300 mg/m^2 ^as a 30-min IV infusion at least one hour prior to RT. This dose was chosen based on literature data [13,15,16] and taking into account that escalating doses of RT would be given. The starting dose of RT was 45 Gy in 25 fractions of 1.8 Gy per day. Dose escalation was achieved by giving 2 fractions of 1.8 Gy per day on a fixed day, with an interfraction interval of at least 6 hours. The escalating dose levels we planned to test were: 54 Gy, 59.4 Gy and 63 Gy. The total duration of the RT was kept on 5 weeks. Five patients were treated in the first dose cohort and followed for one month post treatment before entering patients in subsequent cohorts. We have chosen to escalate the dose by adding an extra fraction of 1.8 Gy 6 to 8 hours after the first one on some days instead of increasing the dose per fraction as smaller fraction sizes induce less late side effects. Moreover, adding extra fractions on some days did not prolong overall treatment time. With this accelerated scheme, we wanted to avoid to prolong the overall treatment time as this may be deleterious (accelerated repopulation) and as these patients have such a poor prognosis that we did not want to jeopardise their limited survival by increasing overall treatment time.

### Radiotherapy

The dose was prescribed at the centre of the target area or at the intersection of central rays of the beam. Highly conformal beams were used, with at least 5 incident beams and 18 MV photons. CT-based treatment planning (with a contrast enhanced CT scan using 5 mm slices) was required for all patients as well as a dynamic MRI in treatment position to assess the corrections needed for breathing movement [17]. The clinical target volume (CTV) included the primary tumour with the peripancreatic and pathological lymph nodes. The patient specific margins around the clinical target volume to account for breathing motion as defined on dynamic MRI were then expanded with another 1 cm to the planning target volume (PTV).

The supportive care program consisted of a close follow up (at least weekly) by a medical doctor combined with a regular consultation with the dietician and prescription of an anti-emetic therapy (5-HT3 antagonist) whenever required.

### Toxicity criteria

Patients interrupted treatment in case of grade 4 adverse events. When one of five patients experienced a dose limiting toxicity (DLT) in a stratum, an additional 5 patients were entered at that dose level. A DLT was defined as one or more of the following events occurring within 8 weeks after the start of treatment: any grade 3 gastro-intestinal (GI) toxicity, any grade 3 liver toxicity that persisted for more than 2 weeks, pancreatitis, any grade 4 skin toxicity within the radiation field, an interruption of the course of RT due to toxicity that lasted more than 2 consecutive weeks, or if 2 interruptions occurred each of which lasted at least one week, an interruption of the course of chemotherapy due to haematological toxicity for 2 consecutive weeks (cycles), febrile neutropenia, or any other grade 3 or 4 toxicity. The RT doses were fixed; however, RT was planned to be interrupted temporarily to manage local toxicity presented in body areas in the radiation volumes, especially in case of grade 3 or 4 adverse events involving small and large intestine. If the toxicity reduced to grade 1 or 2, RT was continued. Based upon the blood counts on the day of treatment, a 20% dose reduction of gemcitabine was given for ANC > 1.0 × 103/L and < 1.499 × 103/L and/or platelet count > 50 × 103/L and < 75 × 103/L. A treatment was dropped for ANC > 0.5 × 103/L and < 0.999 × 103/L and/or platelet count > 20 × 103/L and < 50 × 103/L. A new treatment cycle could begin when blood counts were recuperated. When ANC was < 0.5 × 103/L and/or platelet count < 20 × 103/L, the administration of gemcitabine was stopped.

Patients were examined and toxicities were scored at least every week until 4 weeks after the end of treatment. All toxicities encountered during the course of CRT were evaluated using the RTOG-EORTC Common Toxicity Criteria.

The stratum in which DLT were seen, will be defined as the MTD. The recommended dose of RT was defined as one level below the MTD.

### Surgery

Approximately 4 to 6 weeks after the end of the CRT, patients were re-evaluated with a CT of the abdomen to assess resectability. When a patient had become operable, he/she was referred for surgery. If a patient stayed inoperable, treatment with conventional systemic gemcitabine was given until disease progression or until a total duration of 6 months. The followed scheme was 1000 mg/m^2 ^per week for 3 consecutive weeks with one week of rest.

### Statistical considerations

The study intent was to determinate the DLT of escalating doses of RT delivered concurrently with a fixed dose of gemcitabine (300 mg/m^2^) administered on a weekly basis within the same overall treatment time. The different parameters in both of the dose cohorts were compared by a student's t-test. The relationship between the percentage weight loss and the cumulative GI toxicity (= sum of all different GI toxicities) and the total dose administered to the different normal tissues was evaluated with a Spearman rank correlation test. A p-value ≤ 0.05 was considered to be significant for these tests. Survival was measured from the day of diagnosis until death or the last date of follow up.

## Results

### Patient characteristics and treatment received

Over a 2 year period, 13 patients with locally advanced histologically proven, T3-T4 or stage III pancreatic adenocarcinoma, were enrolled in this study. No patient had received prior therapy for pancreatic cancer. The median age of study participants was 58 years (range, 42–70 years). There were 7 men and 6 women. WHO performance status was 0 in 6, 1 in 5 and 2 in 2 subjects. The median duration of RT was 37 days (range, 32–40 days). The median volume of the PTV was 536.7 cm^3 ^(mean of 522.5 cm^3^), ranging from 200.3 cm^3 ^to 869.0 cm^3^. The volume of each PTV is listed in Table 1 per patient.

**Table 1:** Volume of PTV per patient and the mean dose to the PTV (% of prescribed dose and absolute dose)

	**PTV (planning target volume)**		
**Patient**	**Volume**	**Mean dose %**	**Mean dose Gy**

Patient 1	445.6	99.7	44.9
Patient 2	539.9	100.2	45.1
Patient 3	396	100.1	45.1
Patient 4	590.2	100.4	45.2
Patient 5	637.1	99.1	44.6
Patient 6	200.3	100.5	45.2
Patient 7	318	101	45.5
Patient 8	720.6	101.8	55
Patient 9	419.5	101.7	54.9
Patient 10	536.7	101.9	55
Patient 11	869	100.1	54.1
Patient 12	529.7	100.8	54.4
Patient 13	589.8	99	53.5

Five patients were included in the 45 Gy cohort and they completed the planned treatment without experiencing any DLT. One patient in this cohort required a dose reduction of gemcitabine because of haematological toxicity, but there was no delay in treatment delivery. The next dose level was then tested and 5 patients were included in the 54 Gy cohort. All patients were able to complete the planned treatment, but one patient received only 4 cycles of gemcitabine. Two of 5 patients experienced a DLT, which consisted of acute grade 3 GI toxicity (grade 3 nausea for the first patient and grade 3 nausea and vomiting for the second). An additional 5 patients were planned to enter this dose level, but the first patient also suffered a grade 3 GI toxicity (nausea), so dose escalation was interrupted and the 54 Gy dose level was designated as the MTD and was deemed not suitable for further investigation. Another 2 patients were then studied at the dose level below (45 Gy).

### Toxicity

All 13 patients were evaluable for toxicity analysis, with the different GI toxicities experienced per dose cohort during CRT shown in Table 2. Although it was not the intent of the study to perform a formal comparison between the two dose cohorts (due to the limited number of patients and the non-randomised setting), we report here differences and correlations that might be of interest. Between both dose levels, there was a significant difference in percentage weight loss (4.51% ± 2.11 in the 45 Gy cohort and 11.88% ± 5.26 in the 54 Gy cohort; p = 0.006) and also in cumulative GI toxicity (p = 0.027). There was no grade 3 toxicity in the 45 Gy cohort versus 4 grade 3 toxicity events in the 54 Gy cohort. There was no significant difference in the mean dose to the stomach between both dose levels but the mean dose to the duodenum was significantly higher in the 54 Gy cohort (38.45 Gy vs. 51.82 Gy; p = 0.001).

**Table 2:** GI toxicity: The different GI toxicities experienced (nausea, vomiting and diarrhoea) for each dose cohort.

**Gastro-intestinal (GI) toxicity**						
	**45 Gy cohort (7 patients)**			**54 Gy cohort (6 patients)**		
	**Nausea**	**Vomiting**	**Diarrhoea**	**Nausea**	**Vomiting**	**Diarrhoea**

Grade 0	0/7	1/7	5/7	0/6	0/6	2/6
Grade 1	2/7	4/7	2/7	0/6	3/6	3/6
Grade 2	5/7	2/7	0/7	3/6	2/6	1/6
Grade 3	0/7	0/7	0/7	3/6	1/6	0/6
Grade 4	0/7	0/7	0/7	0/6	0/6	0/6

### Non-resected patients

Eight of the 13 patients did not undergo resection. For three of these patients, a palliative Roux-en-Y choledochojejunostomy and gastrojejunostomy were performed pre-CRT. In one patient, it was performed post-CRT because of the evidence of evolutive disease with a gastric outlet obstruction and in another patient because of persisting inoperability at exploratory laparotomy. In the remaining three patients, there was no possibility for a surgical procedure and two cases continued with gemcitabine according to the study protocol.

### Surgical results

Five of the 13 patients underwent a Whipple procedure. One required a reconstruction of the portal vein; two other procedures required a splenectomy, of which one also included an en bloc resection of the left kidney. There was no postoperative death, and only one patient suffered from a complication (bilateral pneumonia, medically treated). Two patients have no evidence of disease (one patient in the 45 Gy cohort and one patient in 54 Gy cohort). From the three remaining patients in the group with resection, one had an omental metastasis at surgery and received gemcitabine thereafter, and the other two presented with local failure and/or metastases, so a treatment with systemic gemcitabine was started.

### Long-term outcome and survival

The overall median survival for the 13 study patients was 20.3 months. Three of 5 patients in the 45 Gy cohort were operated on, and 2 of 6 in the 54 Gy cohort. The median disease free and 2-year overall survival for the group with resection was 12.6 months and 39%. Three patients are alive at the time of this report and 2 of them have no evidence of disease (1 patient in the 45 Gy cohort and 1 patient in 54 Gy cohort). They both underwent a complete surgical resection. The third patient is currently treated with gemcitabine and has a stable disease.

## Discussion

The primary objective of this trial was to determine the MTD of escalating doses of radiation therapy that could be delivered concurrently with a fixed dose of gemcitabine (300 mg/m^2^), administered on a weekly basis, within the same overall treatment time. We have concluded that a dose escalation to 54 Gy in 1.8 Gy fractions in an accelerated fractionation regimen was the MTD. GI toxicity was the DLT. Therefore we do not recommend this dose for further investigation.

Varying doses and schedules of gemcitabine and concomitant RT for patients with locally advanced pancreatic cancer have been investigated, mainly in phase I clinical trials [16,18-20]. Gemcitabine 300 – 600 mg/m^2^/week given as a once weekly infusion concurrent with conventional RT of 50.4 Gy was reported to be reasonably well tolerated with some indication of anti-tumour activity [13,15,16]. In a phase I study by McGinn et al. to find the MTD of gemcitabine in association with RT to 50.4 Gy in 1.8 Gy daily fractions in patients with locally advanced non-resectable pancreatic cancer, a DLT was reported in 3 of 13 patients [21]. Ten patients did not experience a DLT at the following dose levels: 200 mg/m^2 ^(3 patients), 300 mg/m^2 ^(4 patients) and 400 mg/m^2 ^(3 patients). One patient had a DLT as a result of grade 3 neutropenia at a dose of 300 mg/m^2 ^gemcitabine weekly. The most frequently reported toxicities were GI (nausea and vomiting). Therefore a dose of gemcitabine 300 mg/m^2 ^on a weekly basis was chosen in our study. This was a precaution measure, to take into account the expected additional toxic effects of gemcitabine concurrently with dose-escalated accelerated RT. Despite this, the escalation to a 54 Gy dose level in an accelerated regimen was shown to be not feasible.

McGinn et al. published the results of a phase I trial where the investigators also combined a fixed weekly dose of gemcitabine (1000 mg/m^2^) with an escalating dose of RT [20]. Escalation was achieved by increasing the fraction size in increments of 0.2 Gy, keeping the duration of radiation constant at 3 weeks. The starting dose was 30 Gy (in 2 Gy fractions) and the final dose investigated (42 Gy in 2.8 Gy fractions) was not recommended for further study considering the (potential) occurrence of both acute and late toxicity. As in our study, the acute toxicity consisted of dose-limiting GI toxicity. Application of the linear quadratic model indicates that 42 Gy in 2.8 Gy-fractions is biologically equivalent to 50.4 Gy in 1.8 Gy-fractions, a standard dose and fractionation schedule used in the treatment of patients with unresectable pancreatic cancer. However, a radiation dose of 36 Gy in 2.4 Gy-fractions was well tolerated and this is biologically equivalent to approximately 41.4 Gy in 1.8 Gy-fractions with regard to late effects. Also the radiation field size was defined much smaller than radiation field sizes used in our CRT regimen, because the RT volume is the most critical variable influencing GI toxicity in gemcitabine-based CRT regimens. Nevertheless, McGinn et al. did not report on an excess in local or regional failures by this reduction in radiation dose and field size.

The inclusion of prophylactic nodal basins in the treatment volume, resulting in a large volume of normal tissue irradiated with increased radiosensitization of normal tissues, in combination with the accelerated fractionation and dose escalation, may have led to the toxicity pattern described in our study. Therefore, it is recommended to use guidelines for standardised treatment and volume delineation. Efforts have been put to identify the elective lymphatic target volume in pancreatic cancer and the large topographic variability of upper abdominal lymphatics may have consequences on PTV definition, resulting in an adaptation of the treated volume [22,23]. Also, the adaptation of the PTV according to the dynamic MRI, to account for the potential shift of the target volume due to respiration, has led to an increased radiation volume (mean PTV volume was 522.5 cm^3^). The recommended dose level according to our results is 45 Gy in 1.8 Gy fractions. When dose escalation in an altered fractionation regimen is considered, this has to be performed more conservatively. Perhaps it would have been more feasible if we had given an extra fraction in week 1, 3 and 5 in the first dose escalation cohort to a total dose of 50.4 Gy, followed by an evaluation of this group and then we could have added one fraction per week.

Equally encouraging is the observation that the median survival in this group of patients with a dismal prognosis was 20.3 months, indicating that CRT may play a role in the therapy of borderline resectable or unresectable LAPC. Another important field of interest and future research which may lead to a significant clinical impact on therapy for this poor prognostic cancer, is the combination of molecular targeted agents like nelfinavir [24] or erlotinib, gefitinib and bevacizumab [25-27] with a CRT regimen, although further confirmation of initial positive results in phase I studies is warranted by randomised trials.

## Conclusion

This gemcitabine-based CRT regimen with accelerated dose escalation is clearly not feasible. GI toxicity is the DLT. However, the median survival and the number of inoperable patients becoming resectable, indicate that there might be a role for the investigation of CRT with a fixed dose of gemcitabine and concurrent RT with small fractions (1.8 Gy/day) in borderline resectable or unresectable non-metastatic LAPC.

## Competing interests

The authors declare that they have no competing interests.

## Authors' contributions

All authors read and approved the final manuscript. TB analysed the patient data and drafted the manuscript. KH participated in the study design and the patient evaluation and study enrolment and helped to draft the manuscript. EVC participated in the study design and the patient evaluation and study enrolment and helped to draft the manuscript. WVS participated in the patient evaluation and study enrolment. BT participated in the patient evaluation and study enrolment. RA participated in the patient evaluation and study enrolment. NE participated in the patient evaluation. DB participated in the patient evaluation. DVB participated in the patient evaluation. LG participated in the study design and patient follow up during RT. CV participated in the patient evaluation and study enrolment and helped to draft the manuscript.
